# Prevalence of Intestinal Protozoa and Soil Transmitted Helminths Infections among School Children in Jaragedo Town, South Gondar Zone of Ethiopia

**DOI:** 10.1155/2022/5747978

**Published:** 2022-03-12

**Authors:** Melaku Wale, Solomon Gedefaw

**Affiliations:** ^1^Bahir Dar University, Bahir Dar, Ethiopia; ^2^Koma Secondary School, South Gondar, Ethiopia

## Abstract

**Background:**

Parasitism is a relationship where one, the parasite, harms the host or lives at the expense of the host. Intestinal parasites (protozoa and STHs-soil-transmitted helminths) cause gastrointestinal tract infection in humans and animals. Intestinal parasitic infections (IPIs) predominate the tropics and subtropics and affect poor countries, where school children suffer the most. To prevent and control these infections, local risk factors must first be identified. The aim of the study was to determine the prevalence of intestinal parasitic infections and associated risk factors among school children in Jaragedo town schools, South Gondar Zone of Ethiopia.

**Methods:**

A cross-sectional study was conducted from October 2018 to April 30, 2019, involving 396 students from one elementary and one secondary school. Stratified simple random sampling method was used. A questionnaire was prepared to collect sociodemographic and socioeconomic data of the study subjects. Stool samples were collected and examined using formalin-ether concentration technique. Data were analyzed using SAS software version 9.4. Descriptive statistics were used to give a clear picture of population characteristics. Logistic regression was also used to determine the relationship between dependent variables (primary infection) with independent (explanatory) variables using SAS software.

**Results:**

Results showed that the overall prevalence of intestinal parasitic infections was 65.4%. *E. histolytica* was the most prevalent intestinal parasite (12–14%) followed by *G. lamblia* (8–9%); other parasites could not infect more than 5% of the study subjects. Generally, parasitism did not vary between the sexes. The logistic regression analysis showed that grade, level of students, water source, habit of consuming raw meat, and level of income had a strong effect on intestinal parasitic infection (*P* < 0.05). Other explanatory variables were not significant (*P* > 0.05). High prevalence of parasites indicates improper disposal of waste, low socioeconomic level, low living standard, and poor water quality.

**Conclusion:**

Therefore, short-term and long-term intervention strategies are required to minimize rates of infection.

## 1. Introduction

Parasitism is the relationship in which one of the participants, the parasite, harms or lives at the expense of the host. Parasites may cause mechanical injury, dig into the skin or other tissues of its host, stimulate an inflammatory or immune responses, or simply rob the host of nutrition. Most parasites inflict a combination of those conditions on their hosts [1].

Intestinal parasites infect the gastrointestinal tract and affect health [2]. Parasitic protozoa are single cell microorganisms that possess one and rarely two nuclei. The two most prevalent intestinal protozoa among children are *E. histolytica* and *G. lamblia*. *E. histolytica/dispar* infects 10% of the worlds' population, while the prevalence may rise as high as 50% in many countries [3]. The prevalence of giardiasis was 2 to 7% in developed and 20 to 30% in developing countries. The disparity in prevalence emanates from factors such as geographical area, the urban or rural setting of the society, age, and socioeconomic conditions [4]. Parasitic helminths include nematodes (round worms), trematodes (flukes), and cestodes (tapeworms). The transmission is by contact with water, food, or soil contaminated with infected human feces; these infections cause anemia, vitamin A deficiency, stunted growth, malnutrition, intestinal obstruction, and impaired mental development. The global prevalence of soil-transmitted helminths is high. Recent estimates show that 1472 million people have round worms, 1298 million have hookworms, and 1049 million have whipworm infection [5].

Soil transmitted helminths and protozoan intestinal infections are common among preschool and school-age children. Children tend to play on soil, suck fingers, and defecate in open fields. Maternal awareness also has its own impact on the prevalence. In Ethiopia, poor sanitation and poor personal hygiene and lack of potable water contribute to the high prevalence of parasitism and the associated morbidity and mortality [6]. Playing with soil, sucking fingers, defecation in open fields, lack of potable water, lack of latrines, contact with animals, poor waste disposal system, walking barefoot, poverty, and occupation of family members all contribute to the high parasitism level. Soil transmitted helminths and protozoan intestinal infections are common diseases in Ethiopia. In Jaragedo town, South Gondar zone, Ethiopia, diarrheal cases are common, but the status of school children with respect to helminths and protozoan intestinal infections is unknown, and the current study was conducted to address the gap.

## 2. Materials and Methods

### 2.1. Description of Study Area

The study was conducted in Andabet district, Jaragedo town, South Gondar zone, Amhara region, Ethiopia. Andabet district covers an area of about 8000 ha, and it is located 150 km from Bahir Dar, the regional capital, and 91 km from Debre Tabor, the zonal capital. The total population is 154,797 (51.2% male and 48.8% female). The area receives an annual rainfall of 673.2–1538 mm, temperatures range from 11–25.5°C, and altitudes vary between 1500 and 2300 m above sea level.

The geographical location of Andabet district is 11°10′ to 11°30′ North latitude and 37°45′ to 38°00′ East longitude. In Andabet district, 19 rural and 2 urban kebele administrations are found, one of which is Jaragedo kebele. Jaragedo town has a population of 8894 (5257 males and 3637 females), and it is located 12 km from Andabet town. One-half (50%) of the residents are farmers, 15% merchants, 3% weavers, and 2% others. And 95% of the town dwellers are Orthodox Christian, and 5% are Muslims (Jaragedo community administration, Pers. Comm., 2010). About 66% of the population had access to pipe water.

### 2.2. Study Design

A cross-sectional study was conducted from October 2018 to April 30, 2019, to determine the prevalence of soil-transmitted helminths and protozoan intestinal infections and associated risk factors among school children of Jaragedo primary school and Jaragedo secondary school.

### 2.3. Sample Size Determination and Sampling Technique

#### 2.3.1. Sample Size Determination Technique

A total of 403 (202 females and 201 males) were included in the study. Out of these, 102 male and 101 female students were from the Jaragedo primary school and 100 male and 100 females from Jaragedo secondary school. Fifty-percent prevalence rate was used in the sample size calculation because there was no published report in the study area. The minimum sample size “*n*” required was determined using the statistical formula [7].

(1) $$\begin{array}{c} n=\frac{Z^{2*}p\left(1-p\right)}{m^{2}}, \end{array}$$

where *n* = required sample size, *Z* = standard value of 1.96, *p* = prevalence rate, and *m* = margin of error of 0.05. To minimize errors arising from nonresponse, 5% of the sample was added to the initial estimate. Seven students were excluded from the study because of insufficient data; the total sample size was, therefore, 396 students.

#### 2.3.2. Sampling Technique

Students were stratified according to grade level (1–4, grades 5–8, and 9–10) to select target groups. Quota was allocated for each stratum, grade level, and section. Finally, a systematic random sampling technique was used by using the classroom roster as a sample frame.

### 2.4. Inclusion and Exclusion Criteria

All children registered in both schools and who agreed and signed the consent were included in the study. Students who were treated for intestinal parasites in the last two months and those whose parents were unwilling were excluded.

### 2.5. Data Collection

A structured questionnaire was prepared to collect sociodemographic and socioeconomic data of the study subjects (including risk factors). The questionnaire was first written in English and then translated into Amharic. Students and parents filled the questionnaire. Fingernails of children were inspected. Accuracy and completeness of the filled questionnaire were verified at the end of each day. Immediately after that, each child was given a dry, clean, and leak proof stool cup labeled with the identification number and an applicator stick. Students were advised to fill the stool cup with their own fresh stool about the size of a bean (approximately 3 g) and bring them to the researcher. Stool samples were preserved in 8 ml of 10% formalin solution and transported to Jaragedo Health Center for parasitological examination. A formalin-ether concentration technique was employed to examine the collected samples.

### 2.6. Parasitological Examination

#### 2.6.1. Formalin-Ether Concentration

A formalin-ether concentration technique was adopted. About 1 to 1.5 g of stool specimen was mixed in a centrifuge tube containing 10 ml formalin mixture and stirred until a suspension formed. Then, 3 ml of ether was added to the suspension and thoroughly mixed by putting a rubber stopper in the tube and then shaken for ten seconds. The tube was placed in a centrifuge for 2–3 minutes at 2000 revolutions per minute. Then, the tube was removed from the centrifuge, where 4 layers have been observed from the top to the bottom (top layer ether, 2^nd^ layer fat debris, 3^rd^ layer formalin, and bottom layer sediment). The first three layers were discarded. A small amount of residual fluid was flown back to the sediment, properly mixed with the sediment, and a drop of suspension was transferred to a clean slide and covered with a cover slip. Finally, the slide was examined at 10x and 40x objectives for the presence of intestinal parasites.

### 2.7. Data Analysis

Questionnaires and parasitological data were analyzed using SAS software version 9.4. Descriptive statistics were used to show population characteristics, i.e., age, sex, prevalence of intestinal protozoa, and soil-transmitted helminthic infections. Logistic regression was used to determine the relationship between dependent variables (primarily infections) with independent (explanatory) variables or risk factors by using SAS software. Parameter estimates were considered significant at *α* (alpha) = 0.05. Mother's level of education and hand washing habits of the study subjects before the meal were excluded from the logistic regression analysis because the sample size was not balanced; i.e., three study participants did not wash hands. Study subjects whose parents had college education were few, and this factor was excluded from the model.

### 2.8. Ethics Clearance

The study was conducted after research ethics approval was obtained from Bahir Dar University, Science College, Bahir Dar, Ethiopia. Letters were written to schools, to district Health Office, and to Jaragedo Health Center. Participants were briefed about the objectives of the study and that all personal information was treated strictly confidential. Consent letter was prepared and ratified with students and parents. Study participants confirmed positive for intestinal parasites were treated free of charge.

## 3. Results

### 3.1. Demographic Characteristics of Study Subjects

Four hundred and six kids were selected for the study. Seven of them (∼2%) were excluded because of lack of stool samples. A total of 396 students took part in the investigation; 197 (49.7%) were male, and 199 (50.3%) were female. About 40% of the students were 17 years or older; 55% resided in rural and the rest in urban areas; 22% were in the first cycle of elementary school (1–4 grades), 28% were in the second cycle of elementary school (5–8 grades), and 50% students were from Jaragedo secondary school.

### 3.2. Risk Factors Associated with Intestinal Parasitic Infections

#### 3.2.1. Socioeconomic Risk Factors

Over 65% of the parents were illiterate, and most fathers were farmers. About half of the parents (∼48%) used both tap water and river water interchangeably; 38% of them used tap water alone. Over 70% of them belonged to the middle-income group; the rest were rich or poor. About 23% of the families involved had a family size less than 5, 60% had 5 to 6 members, and 20% above 7.

#### 3.2.2. Behavioral Risk Factors

Over 90% of the study subjects washed hands before meal and after toilet use; more than 75% of them washed vegetables. Slightly more than half of the kids did not consume raw meat. Most of the students (∼85%) wore shoes always. Over 90% of families had their own toilet in close proximity, and more than 80% of them buried household waste. On direct observation, about 35% of the students had unclean nails. The details are given in Table 1.

### 3.3. Prevalence of Intestinal Parasitic Infections

**Figure 1:**
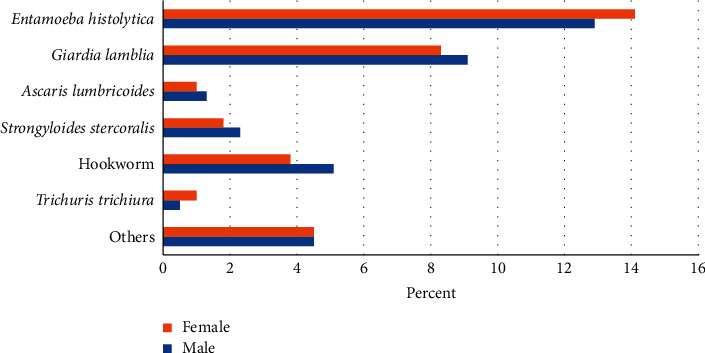
Prevalence of single intestinal parasites among school children in Jaragedo primary and secondary schools, northwest Ethiopia.

Overall prevalence of intestinal parasitic infections was 65.4%. *E. histolytica* was the most prevalent intestinal parasite (12–14%) followed by *G. lamblia* (8–9%); other parasites, the ones that could not be grouped into any of the six species, infected less than 5% of the study subjects. Parasitism did not vary between the sexes (a difference of only about 2%) (Figure 1).

As the odds ratios show, the prevalence of intestinal parasitic infections was high for students who were 15-16 years old, grades 9-10, rural dwellers, illiterate family heads, families who practiced farming, very poor families, river water users, and students with six family members (Table 2). Kids who did not wash hands before the meal and who did not wear shoes were all found infected. The odds of being positive for intestinal parasitic infections were about twice higher among rural dwellers compared to urban dwellers. Students who did not wash their hands after use of the toilet had two times higher odds ratio having these infections compared to students who washed hands. Students from very poor families were 5 times more likely to be infected compared to kids from rich families; those who did not use latrines had two-fold chance of being affected compared to children who used latrines. Increasing level of education of fathers helped reduce infection compared to fathers without education. River water users were 7.5 times more likely to be infected compared to tap water users. The odds of being positive for intestinal parasitic infections were two times higher among kids in 15–16 age category compared to kids in 7–10 age categories. Secondary school grade levels were 2.5 times more likely to be infected compared to elementary school grade levels.

A large majority of the students were found infected by a single parasite (61%), and only 4% had double infection. Those who consumed unwashed vegetables, whose nails were not clean, who disposed waste in open fields, and who had no latrine were found more likely to be infected.

### 3.4. Logistic Regression Analysis

#### 3.4.1. Protozoan Infections

About 44% of the subjects were found positive for *E. histolytica/dispar* or *G. lamblia* or both. According to the results of the logistic regression analysis, father's level of education, consumption of raw meat, source of water, and level of income had a strong effect on protozoan infection (*P* < 0.05) (Table 3). Other explanatory variables were not statistically significant (*P* > 0.05).

#### 3.4.2. E. histolytica/Dispar

*E. histolytica/dispar* was the leading parasite identified among the school children under investigation. According to logistic regression analysis father's occupation, water source, family size, and consumption of raw meat had a strong effect on *E. histolytica/dispar* infection (*P* < 0.05) (Table 4). Other explanatory variables remained statistically insignificant (*P* > 0.05).

#### 3.4.3. *G. lamblia*

*G. lamblia* was the second most prevalent intestinal parasite. Logistic regression analysis showed that water source was the only explanatory variable that is significantly associated with *G. lamblia* infection (*P*=0.0059) (Table 5). Other variables were not significant (*P* > 0.05).

#### 3.4.4. Soil Transmitted Helminths

Out of the total study participants, 16.7% confirmed positive for soil-transmitted helminthic parasites. From the logistic regression analysis, age of study subjects, habit of shoe wearing, water source, waste disposal system of families, latrine type, and accessibility had a strong effect on soil-transmitted helminthic infection (Table 6).

#### 3.4.5. *Ascaris lumbricoides, Strongyloides stercoralis,* and *Trichuris trichiura* Infections

*A. lumbricoides, S. stercoralis,* and *T. trichiura* occurred rarely. They were, therefore, less important in the current study.

#### 3.4.6. Hookworm Infection

Hookworm infection was the third most prevalent species of parasites among school kids in Jaragedo town. The logistic regression showed significant association with wearing shoes, water source, and access to latrine (*P* < 0.05).

#### 3.4.7. Other Intestinal Parasites

During the parasitological examination, other intestinal parasites that could not be grouped to any of the six parasites were found in the stool samples in addition to the six species. Their overall prevalence was 9.1%. The logistic regression analysis showed that only water source had a strong effect on these other intestinal parasitic infections (*P* < 0.05).

#### 3.4.8. All Intestinal Parasites

The overall prevalence of intestinal parasitic infections in the current investigation was 65.7% including all gastrointestinal parasitic species. The logistic regression analysis showed that grade level of students, water source, habit of consuming raw meat, and level of income were significantly associated with all intestinal parasites taken together.

## 4. Discussion

The overall prevalence of intestinal parasitic infection among school children of Jaragedo town was 65%, which corroborates previous reports in Tigray, Ethiopia (61%) [8], South Africa (65%) [9], Bahir Dar, Ethiopia [10], Sao Tome and Principe (65%) [11], and Borena, Ethiopia (59%) [6].

However, the present prevalence rate was far lower than the results from southwestern Ethiopia (83%) [12], Delgi area of the Amhara region, Ethiopia (78%) [13], and Sudan (84%) [14].

Still, others have reported lower rates than the present figures. Some report a 51% prevalence rate in Adigrat town of Ethiopia [15], 53% in Nigeria [16], 40% in India [17], 28% in Arba Minch town, Ethiopia [18], 44% in Tilili, Ethiopia [19], and 40% in Gamo area, South Ethiopia [20], all studied on children.

In the present study, the overall prevalence of intestinal protozoa infections (*E. histolytica/dispar* and *G. lamblia*) was 44%. This corroborates a study conducted in Mekelle city of Ethiopia among patients with watery diarrhea (45%) [21], Tanzania (48.7%) [22], and Merhabete of Ethiopia (47%) [23]. Other studies reported lower protozoan infections such as Wollo, Ethiopia (11%) [24], Nepal (18.5%) [25], Gerbe Guracha, Ethiopia (17%) [26], and southern Thailand (4.6%) [27].

Contributing factors for the present study could be consuming unwashed vegetables, reluctance to wash hands before meal and after the toilet, accessibility of latrine, eating raw meat, and dirty fingernails.

Soil transmitted infections in the present study gave an overall prevalence rate of 16.7%, which corroborated previous studies: Gelemso, western Hararghe (17.1%) [28], and Gerbe Guracha, Ethiopia (15%) [29]. All of these other reports were conducted on children.

Soil transmitted infections were low in other reports such as Babile town, Ethiopia (<1%) [30], Enderta, Ethiopia (7%) [31], Sao Tome and Principe (11%) [11], and Ambo town, Ethiopia (13%) [32]. The present study gave a lower prevalence of soil-transmitted helminth infections than reports from other areas in Ethiopia such as Jima zone around Gilgel Gibe Dam (44%) among the general community [33], and Jima Arjo (46.7%) among school children [34]. Differences could emanate from variations in the habit of wearing shoes, sources of drinking water, climate, socioeconomic status, habit of clipping and cleaning fingernails, habit of eating raw/unwashed vegetables, waste disposal systems, parasitological methods, and other unknown/undiscovered risk factors.

From the six species of intestinal parasites, the leading one was *E. histolytica/dispar* with a 27% prevalence rate, a report corroborating reports from other regions of Ethiopia such as North Gondar (27%) [35], Berhe et al. (24.7%) [21]. Rituparna et al. [17] reported the overall prevalence of 24% in India. Others reported much lower rates than the current one: Dessie, Ethiopia (6.5%) [36], Nigeria (10.5%) [16], Gerbe Guracha, Ethiopia (9.4%) community-based study [26], Arba Minch town, Ethiopia (12.9%) [18], and Adigrat town, Ethiopia (4.5%) [15]. A study from Haramaya, Ethiopia, reported a 47% infection rate among asymptomatic food handlers working at Haramaya University Cafe [37].

In the present study, the second leading intestinal parasite was *G. lamblia* (17.5%), which corroborated results from Tanzania (16.4%) [22] and Uganda (16.0%) [38]. It was much lower than reports from Delgi, North Gondar, Ethiopia (16.4%) [35], Turkey (47.9%) [39], Sudan (46.9%) [14], and Nepal (40.5%) [17].

On the other hand, the prevalence of *G. lamblia* was 3% in Taif, Saudi Arabia [40], 9.4% in Gondar, Ethiopia, among kids under the age of five [41], 2.3% in Adigrat town, Ethiopia [15], 4.2% in Arba Minch town, Ethiopia [18], and 9.6% in Addis Ababa, Ethiopia, among street dwellers [42, 43].

Hookworm prevalence was 8.8% in the current study. Other reports on hookworm include an estimated Ethiopian national prevalence of 16% [44] and 47% in Sub-Saharan Africa [45]. Lower prevalence of hookworm includes 2.2% in Chuahit, North Gondar, Ethiopia [46].

The overall prevalence of *S. stercoralis* (4.0%) in the current study was higher than 0.21% reported from Enderta area of Ethiopia [31] and 8.6% from Mozambique [47].

The overall prevalence of *A. lumbricoides* in this study was 2.3%, which was similar to other reports such as 3.7% in Slovakia [48]. Another study conducted in Qatar among newly arrived immigrants revealed that the overall prevalence was 1.8% [49]. In contrast, the prevalence of *A. lumbricoides* infection was reported to be 29% in the highlands, 35% in temperate areas, and 38% in the lowlands of Ethiopia [50], 22.1% in Jima [34], and 29% in South Africa [9].

The lowest encountered intestinal parasite was *T. trichiura* (1.5%). This was consistent with the report from Qatar (1.4%) [49], Nepal (1.3%) [51], Chuahit, North Gondar, Ethiopia (1.7%) [46], Nigeria (1.7%) [52], and Merhabete, Ethiopia (1.4%) [28]. Higher rates than the present one include reports from Addis Ababa (22.8%) on street dwellers [42], and Nigeria (16.0%) among elementary school children [53].

In the current study, logistic regression showed that sexes were not associated with intestinal parasites. Hailegebriel [10] has reported similar results before from Bahir Dar area of Ethiopia. In Iran, on the other hand, male gender was significantly associated with intestinal parasites [54]. Using river water was significantly associated with these parasites, as was reported before in Ethiopia from North Gondar [35] and from Arba Minch town [18]. Tap water is safer as it may be chlorinated, while river water gets contaminated with animals and humans. Income was another predisposing factor, as poverty may lead to an unhygienic environment, lack of clean water, improper clothing, and poor nutrition [39]. Raw meat consumption also significantly contributed in a similar manner reported before [35].

## 5. Conclusion

Intestinal parasitic infections were confirmed as common health problems among the Jaragego town school-age children in Ethiopia. *Entamoeba histolytica/dispar*, *G. lamblia,* and hookworm infections were the most common intestinal parasites. High prevalence of these parasites indicates poor disposal of waste, poverty, contaminated water supply, consuming raw meat, etc. Deworming programs, sanitation of the school environment, latrines for males and females, improving water supply, and education of the general community should be launched.

## Figures and Tables

**Table 1:** Behavioral risk factors associated with intestinal parasitic infections.

Risk factors	Options	Frequency (#)	Percent
Hand washing before meal	Yes	393	99.2
	No	3	0.8
Washing hands after toilet	Yes	365	92.2
	No	31	7.8
Do you wash vegetables?	Yes	309	78.0
	No	87	22.0
Do you eat raw meat?	Yes	183	46.2
	No	213	53.8
How often you shoe?	No at all	25	6.3
	Some times	28	7.1
	Always	343	86.6
Way of disposing waste	Burry	326	82.3
	Open space	70	17.7
Latrine type of families	Private	366	92.4
	Common	12	3.0
	Open space	18	4.6
Students' fingernail during interview	Clean	258	65.2
	Not clean	138	34.8

**Table 2:** Prevalence of intestinal parasitic infections among school children in relation to explanatory variables.

Characteristics	Options	Examined	Positive	Negative	Ratio	Odds ratio
Sex	Male	197	130	67	1.94	1.05
	Female	199	129	70	1.84	1
Age (years)	7–10	60	36	30	1.2	1.14
	11–14	93	44	49	0.89	1
	15–16	84	60	24	2.5	2.8
	≥17	159	119	140	0.85	0.95
Residence	Rural	221	159	62	2.56	1.92
	Urban	175	100	75	1.33	1
Education (grade level)	1–4	88	50	38	1.3	0.8
	5–8	109	56	53	1.05	1
	9–10	199	153	46	3.32	3.16
Father's education level	Illiterate	269	185	84	2.2	2.75
	Church	31	20	11	1.8	2.25
	Primary	74	44	30	1.5	1.87
	Secondary	13	6	7	0.85	1.06
	College and above	9	4	5	0.8	1
Father's occupation	Farming	290	198	92	2.15	1.59
	Nonfarming	106	61	45	1.35	1
Water source	Tap	150	74	76	0.97	1
	River	58	51	7	7.28	7.5
	Both	188	134	54	2.48	2.5
Wealth status	Very poor	11	10	1	10	5.2
	Poor	32	19	13	1.46	0.7
	Middle income	295	192	103	1.86	0.97
	Rich	58	38	20	1.9	1
Family size (#)	≥7	75	45	30	1.5	0.78
	6	115	82	33	2.48	1.3
	5	116	73	43	1.6	0.84
	<5	90	59	31	1.9	1
Hand washing after toilet	Yes	365	235	130	1.8	1
	No	31	24	7	3.42	1.9
Washing vegetables	Yes	309	207	102	2.03	1
	No	87	52	35	1.48	0.73
Eating raw meat	Yes	183	111	72	1.54	0.67
	No	213	148	65	2.27	1
Shoe wearing habit	Not at all	25	25	0	—	—
	Sometimes	28	25	3	8.33	5.33
	Always	343	209	134	1.55	1
Waste disposal	Buried	326	208	118	1.76	1
	Open space	70	51	20	2.55	1.44
Type of latrine used by the family	Private	366	236	130	1.8	1
	Common	12	9	3	3	1.66
	Open space	18	14	4	3.5	1.94
Cleanliness of fingernails	Clean	258	162	96	1.68	1
	Not clean	138	97	41	2.36	1.4

**Table 3:** Relationship between intestinal protozoan parasitic infections and explanatory variables among school children in Jaragedo town, South Gondar Zone, Ethiopia.

Term	Parameter estimate	Standard error	*χ* ^2^	*P* value
Intercept	0.317	0.491	0.42	0.518
Age (11 to 14)	0.433	0.284	2.32	0.127
Age (15 to 16)	−0.309	0.300	1.07	0.301
Age (17 and above)	0.320	0.367	0.76	0.382
Sex (female)	0.056	0.117	0.23	0.629
Residence (rural)	0.123	0.152	0.66	0.417
Grade (1 to 4)	0.542	0.415	1.70	0.191
Grade (5 to 8)	0.190	0.242	0.61	0.433
Father education level (Church education)	−0.329	0.439	0.56	0.452
Father education level (college and above)	1.119	0.927	1.46	0.227
Father education level ( illiterate)	−0.679	0.325	4.35	0.036
Father education level (primary education)	−0.036	0.354	0.01	0.917
Father's occupation (farming)	0.120	0.167	0.52	0.471
Hand washing after toile (no)	−0.166	0.244	0.46	0.496
Eating unwashed vegetable (no)	0.149	0.154	0.93	0.334
Eating raw meat (no)	−0.347	0.128	7.33	0.006
Shoe wearing (always)	−0.421	0.249	2.85	0.091
Shoe wearing (no at all)	−0.088	0.354	0.06	0.803
Water source (both tap and river)	−0.295	0.174	2.87	0.090
Water source (river)	−0.875	0.254	11.84	<0.0001
Family economic status (middle-income)	0.484	0.255	3.60	0.057
Family economic status (poor)	0.453	0.377	1.44	0.229
Family economic status (rich)	0.179	0.322	0.31	0.578
Family size (5 members)	0.324	0.200	2.62	0.105
Family size (6 members)	−0.270	0.192	1.97	0.160
Family size (7 and above)	−0.121	0.225	0.29	0.589
Waste disposal system ( burry underground)	0.281	0.176	2.56	0.109
Toilet type (common pit latrine)	0.098	0.562	0.03	0.860
Toilet type (open space)	−0.170	0.462	0.14	0.711
Nail cleanness (not trimmed and not clean)	−0.030	0.131	0.05	0.815

**Table 4:** Relationship between explanatory variables and *Entamoeba histolytica/dispar* infection among school children in Jaragedo town, South Gondar Zone, Ethiopia.

Term	Parameter estimate	Standard error	*χ* ^2^	*P* value
Intercept	0.490	0.496	0.98	0.3229
Age (11 to 14)	0.330	0.310	1.13	0.2877
Age (15 to 16)	−0.141	0.330	0.18	0.6680
Age (17 and above)	0.082	0.391	0.04	0.8340
Sex (female)	−0.017	0.125	0.02	0.8864
Residence (rural)	0.164	0.163	1.01	0.3140
Grade (1 to 4)	0.194	0.455	0.18	0.6690
Grade (5 to 8)	0.167	0.263	0.41	0.5238
Father education level(Church education)	0.122	0.466	0.07	0.7938
Father education level (college and above)	0.779	0.938	0.69	0.4064
Father education level (illiterate)	−0.287	0.340	0.71	0.3981
Father education level (primary education)	−0.127	0.370	0.12	0.7296
Father's occupation (farming)	0.136	0.180	0.57	0.4485
Hand washing after toile (no)	−0.535	0.240	4.95	0.0262
Eating unwashed vegetable (no)	0.148	0.166	0.80	0.3722
Eating raw meat (no)	−0.411	0.137	8.96	0.0028
Shoe wearing (always)	−0.081	0.257	0.10	0.7523
Shoe wearing (no at all)	0.044	0.378	0.01	0.9068
Water source (both tap and river)	−0.443	0.181	5.99	0.0144
Water source (river)	−0.418	0.251	2.76	0.0967
Family economic status (middle income)	0.421	0.258	2.66	0.1026
Family economic status (poor)	0.382	0.383	1.00	0.3178
Family economic status (rich)	0.418	0.342	1.49	0.2217
Family size (5 members)	0.339	0.218	2.42	0.1199
Family size (6 members)	−0.451	0.201	5.04	0.0248
Family size (7 and above)	0.076	0.246	0.10	0.7553
Waste disposal system ( burry underground)	0.142	0.180	0.62	0.4302
Toilet type (common pit latrine)	0.162	0.561	0.08	0.7727
Toilet type (open space)	−0.257	0.464	0.31	0.5799
Nail cleanness (not clean)	−0.020	0.140	0.02	0.8835

**Table 5:** Relationship between explanatory variables and *G. lamblia* infection among school children in Jaragedo town, South Gondar Zone, Ethiopia.

Term	Parameter estimate	Standard error	*χ* ^2^	*P* value
Intercept	3.945	6.589	0.36	0.5494
Age (11 to 14)	0.310	0.392	0.63	0.4287
Age (15 to 16)	−0.26039	0.397	0.43	0.5118
Age (17 and above)	0.190	0.468	0.16	0.6846
Sex (female)	0.094	0.146	0.42	0.5183
Residence (rural)	−0.024	0.194	0.02	0.8998
Grade (1 to 4)	0.625	0.606	1.06	0.3027
Grade (5 to 8)	0.023	0.341	0.00	0.9445
Father education level (Church education)	−2.188	6.578	0.11	0.7394
Father education level (college and above)	5.353	26.24	0.04	0.8384
Father education level (illiterate)	−1.927	6.569	0.09	0.7692
Father education level (primary education)	−0.868	6.577	0.02	0.8950
Father's occupation (farming)	−0.020	0.235	0.01	0.9289
Hand washing after toile (no)	0.354	0.315	1.26	0.2609
Eating unwashed vegetable (no)	0.058	0.191	0.09	0.7593
Eating raw meat (no)	0.065	0.156	0.17	0.6760
Shoe wearing (always)	−0.580	0.374	2.40	0.1210
Shoe wearing (no at all)	−0.248	0.491	0.26	0.6131
Water source (both tap and river)	0.089	0.203	0.19	0.6607
Water source (river)	−0.743	0.269	7.59	0.0059
Family economic status (middle income)	0.286	0.308	0.86	0.3531
Family economic status (poor)	0.383	0.472	0.66	0.4167
Family economic status (rich)	−0.362	0.382	0.90	0.3418
Family size (5 members)	0.122	0.242	0.25	0.6137
Family size (6 members)	0.231	0.243	0.90	0.3424
Family size (7 and above)	−0.263	0.277	0.90	0.3430
Waste disposal system ( burry underground)	0.177	0.206	0.74	0.3904
Toilet type (common pit latrine)	0.488	0.839	0.34	0.5605
Toilet type (open space)	−0.480	0.608	0.62	0.4299
Nail cleanness (not clean)	−0.031	0.157	0.04	0.8399

**Table 6:** Relationship between explanatory variables and soil-transmitted helminthic infections among school children in Jaragedo town, South Gondar Zone, Ethiopia.

Term	Parameter estimate	Standard error	*χ* ^2^	*P* value
Intercept	0.388	0.599	0.42	0.5172
Age (11 to 14)	−0.769	0.467	2.71	0.0994
Age (15 to 16)	0.308	0.499	0.38	0.5376
Age (17 and above)	1.120	0.544	4.23	0.0397
Sex (female)	0.050	0.173	0.09	0.7702
Residence (rural)	−0.377	0.220	2.93	0.0870
Grade (1 to 4)	0.514	0.600	0.73	0.3919
Grade (5 to 8)	0.450	0.364	1.53	0.2156
Father education level (Church education)	−0.211	0.581	0.13	0.7155
Father education level (college and above)	−0.903	0.984	0.84	0.3591
Father education level (illiterate)	0.703	0.429	2.68	0.1016
Father education level (primary education)	−0.331	0.445	0.56	0.4560
Father's occupation (farming)	−0.236	0.262	0.81	0.3680
Hand washing after toile (no)	−0.127	0.344	0.14	0.7104
Eating unwashed vegetable (no)	0.146	0.226	0.42	0.5166
Eating raw meat (no)	0.125	0.184	0.46	0.4959
Shoe wearing (always)	2.276	0.316	51.67	<0.0001
Shoe wearing (no at all)	−1.588	0.402	15.61	<0.0001
Water source (both tap and river)	0.218	0.243	0.81	0.3691
Water source (river)	−0.706	0.328	4.63	0.0314
Family economic status (middle income)	−0.146	0.360	0.17	0.6836
Family economic status (poor)	0.039	0.560	0.01	0.9431
Family economic status (rich)	−0.463	0.460	1.01	0.3142
Family size (5 members)	−0.057	0.290	0.04	0.8425
Family size (6 members)	−0.126	0.285	0.20	0.6582
Family size (7 and above)	0.351	0.365	0.92	0.3364
Waste disposal system (burry underground)	−0.658	0.302	4.72	0.0298
Toilet type (common pit latrine)	−1.167	0.582	4.02	0.0450
Toilet type (open space)	1.194	0.651	3.37	0.0666
Nail cleanness (not clean)	−0.304	0.192	2.50	0.1135

## Data Availability

Data are available from the corresponding author upon request.
